# Is the Indonesian disaster response budget correlated with disaster risk?

**DOI:** 10.4102/jamba.v11i1.759

**Published:** 2019-11-14

**Authors:** Heru Fahlevi, Mirna Indriani, Rina S. Oktari

**Affiliations:** 1 Faculty of Economics and Business, Universitas Syiah Kuala, Banda Aceh, Indonesia; 2 Tsunami and Disaster Mitigation Research Center, Universitas Syiah Kuala, Banda Aceh, Indonesia; 3 Faculty of Medicine, Universitas Syiah Kuala, Banda Aceh, Indonesia

## Abstract

This study investigates the correlation between disaster budget, index of disaster risks and the population of 23 districts in the Aceh province, northern Indonesia. It also explores how the budget for disasters is proposed and prepared by Indonesian local governments. A descriptive quantitative approach is used to examine this relationship. Data were collected from the budgets of local governments (*Badan Penanggulangan Bencana Daerah* [BPBD] or local disaster management agency) and the disaster risk index. In addition, surveys were undertaken in which the respondents were the key officers in the BPBDs who are responsible for the budget and programmes to obtain detailed information about how the local government prepares their budget for disaster. By employing the Pearson’s chi-square test and the Pearson correlation test, this study revealed no significant statistical relationship between the disaster budget and the level of disaster risks among districts or cities in the Aceh province, northern Indonesia. However, results show that the total budget of the local governments has a significant positive correlation with the disaster budget. The surveys also confirm the correlation between the total budget of the local government and disasters as the same budgeting procedure is applied. The result generalisability might be limited as this study only focused on one of Indonesia’s provinces, Aceh.

**Keywords:**

disaster risk; disaster relief planning; disaster budget; local government; tsunami; Indonesia.

## Introduction

Indonesia is well known for high disaster risks, particularly over the last two decades. Its demographic, geological and geographical situations could be the main reasons why the country with a population of more than 240 million experiences various disasters. The country is situated in the highest disaster risk area known as the Pacific Ring of Fire where the three tectonic plates of Indo-Australia, Eurasia and the Pacific are located (Australian Broadcasting Corporation International Development 2017). Consequently, there are approximately 20 earthquakes per day recorded within the Indonesia region (Australian Broadcasting Corporation International Development [ABCID] 2017). It is not only the earthquakes but also other natural disasters such as landslides, forest fires, floods and volcano eruptions that are frequently faced by the Indonesian people. These disasters cost the lives of many people and severely affect the economy across the domestic regions. Phaup and Kirschner (2010) report that Indonesia experienced infrastructure losses of approximately $4.5 billion in the Indian Ocean earthquake and tsunami of 2004.

To mitigate and reduce the large-scale disaster impact on the socio-economic situation, a disaster risk reduction (DRR) programme has been called for by experts and the international community (Wada et al. 2014). More importantly, the integrated DRR programme could potentially reduce the possibility of human losses. In this context, any government should implement a more preventive approach rather than solely focusing on the responsive and emergency actions (De La Fuente 2010). This includes the allocation of an adequately sized disaster budget (DB) for a preventive disaster mitigation programme (Oktari, Fahlevi & Irawati 2017).

Several authors find that developing and low- or middle-income countries are more likely to be impacted by disaster than developed countries (see, for instance Fankhauser & McDermott 2014; Loayza et al. 2012; Toya & Skidmore 2007). In terms of the economy, Indonesia is a developing country in which disasters can immediately damage the country and the financial conditions of victims, especially the poor people. The enactment of Law No. 24 of 2007 on disaster management (DM) has shifted the disaster paradigm and brought a new concept of DRR in Indonesia. The focus of DM was previously on emergency response and was considered as an extraordinary effort, which was the responsibility of the government. The focus has now shifted to risk management, where DRR is part of daily routine activities and becomes an important element of development agendas which need the involvement of the community, non-governmental organisations (NGOs) and the private sector. Since 2007, DRR in Indonesia is considered as a structural, planned and sustainable effort. However, several researchers argue that the DRR concept in Indonesia has not been fully and institutionally considered or coupled in the government structures including budgeting, especially at the sub-national levels (Djalante et al. 2015).

In general, the DRR investment ratio of the Indonesian government is still lower than the internationally agreed ratio. Darwanto (2012) documents a relatively smaller DRR investment ratio for the Indonesian government compared to the international standard, namely 1% of the total national budget. In addition, the DRR programme is not a priority of local governments as is reflected by a small budget allocation for disaster-related programmes. For example, Oktari et al. (2017) reveal that the government of Banda Aceh, a city that was destroyed by the 2004 Tsunami, allocated an insignificant amount of its budget for disaster-related programmes and the budget portion has remained the same over the years.

Moreover, determining how much financial loss results from a natural disaster is not an easy task and budgeting in advance for a disaster could be far more complicated. *Ex-ante* budgeting may lead to preventive strategies that are more effective and to the promotion of a disaster mitigation programme (Dartanto, Bastiyan & Sofiyandi 2017). However, budgeting for disaster mitigation programmes can have several major challenges, that is, political incentives to delay the recognition of costs for a DB until the disaster happens; moral hazard and the belief that it is impossible or at least difficult to save government money for disasters (Phaup & Kirschner 2010). Notwithstanding, this many budgets for disasters or expenditures are not easy to be traced as they are indirectly contributed to disaster mitigation and prevention (De La Fuente 2010).

To our knowledge, research on disaster budgeting is still scant (Oktari et al. 2017). Most of the previous research deals with the calculation of financial loss caused by disasters and government spending for disasters (see, e.g. Benali, Abdelkafi & Feki 2018; Toya & Skidmore 2007). Indeed, previous studies such as by Kusumasari, Alam and Siddiqui (2010) have been conducted mostly in developed countries rather than in developing countries. Hence, the present study analyses the association between the DB, index of disaster risks and the population of 23 districts in the Aceh province, northern Indonesia, between 2014 and 2016. Aceh is selected as the site of study because this region was partly destroyed during the 2004 tsunami and other disasters, that is, earthquakes and floods. The present study assumes that the vulnerability of the region to natural disasters has been (or should have been) taken into account in allocating budget for disasters. The DB is represented by how much money is provided annually for *Badan Penanggulangan Bencana Daerah* (BPBD or local DM agency) as the government department responsible for the DM. In addition, this study is conducted at the local government level because they know more about their citizens and situation compared to other actors, that is, central government (Kusumasari et al. 2010; Putra & Matsuyuki 2019).

## Disaster management system in Indonesia

The DM system in Indonesia has been changed substantially since a few decades ago from the centralised and reactive to the more decentralised, preventive and integrative approach that involves both national and sub-national levels and other non-state actors (Nugraha & Lassa 2018). These changes can be associated with the paradigm shift for disaster and decentralisation reform.

Later on, the decentralisation reform transformed the Indonesian government, administrative and fiscal structures. Since 1998, the decentralisation process has been started with some barriers and challenges. Many authors (e.g. Alm, Aten & Bahl 2001; Das & Luthfi 2017; Fahlevi 2016) argue that the decentralisation reform is ambitious and partially implemented, including the DM system. After the enactment of Indonesian Law No. 24/2007 on DM, the government of Indonesia embraced a more comprehensive and extended version of the DM framework and sharing responsibilities between the central government and local governments. However, the Indonesian government apparently does not have a cost and financing sharing plan for a DM programme that inclusively integrates any contributions from the central and local governments.

The Law 24/2007 becomes the entry point of the disaster paradigm shift from reactive emergency response to pro-active strategies in DM. The law has provided a legal arrangement for programme implementation to protect the community at all stages of pre-, during and post-DM as briefly described in Table 1.

**TABLE 1:** Pre-disaster programme activities mandated by the Indonesian Disaster Management Law 24/2007.

Programme	Activities	Source
**A. Pre-disaster situation where there is no disaster**		**Article 35**
Disaster management planning	(1) Identification and assessment of hazard, (2) community vulnerability assessment, (3) analysis of disaster potential impact, (4) disaster risk reduction measurement, (5) disaster preparedness and response mechanism and (6) allocation of tasks, authority and available resources	Article 36, (4)
Disaster risk reduction	(1) Disaster risk identification and monitoring, (2) participatory disaster management planning, (3) promotion of disaster-awareness practices, (4) increase commitment of disaster management practitioner and (5) application of physical and non-physical efforts and instructions on disaster management	Article 37, (2)
Prevention	(1) Identification and knowledge of hazards sources, (2) natural resources management of what can potentially become a disaster, (3) monitoring the use of technology of what can potentially become a source of disaster, (4) spatial planning and environmental management and (5) strengthening of community resilience	Article 38
Integration into development planning	Disaster management plan elements to be included in national and regional development plans	Article 39
Disaster risk analysis requirements	(1) Prepare and stipulate disaster risk analysis requirements, (2) fulfilment of disaster risk analysis requirements shown in a document ratified by a government official in accordance with legislation and (3) carry out the risk analysis monitoring and evaluation	Article 41, (1)(2)(3)
Implementation and enforcement of spatial planning	(1) Regulations on spatial planning and safety standards impose a sanction against violators and (2) monitor and evaluate periodically the implementation of the spatial plan and achievement of safety standards	Article 42, verse (1)( 2)
Education and training	Carry out and stipulate education and training requirements for disaster management in accordance with legislation	Article 43
Technical standard requirements for disaster management	Perform and stipulate technical standard requirements for disaster management in accordance with legislation	Article 43
**B. Pre-disaster situation regarding potential disaster**		**Article 44**
Preparedness	(1) Preparation and try-out for contingency plans, (2) organisation, installation and testing of early warning system, (3) provision and preparation of basic needs supplies, (4) organisation, counselling, training and drill regarding emergency response mechanism, (5) preparation of evacuation location, (6) development of accurate data, information and update on emergency response standard operational procedures and (7) provision and preparation of materials, goods and equipment for facilities and infrastructure recovery	Article 45, (2)
Early warning	(1) Observation of disaster signs, (2) analysis from disaster signs observation results, (3) decision-making by the authorities, (4) dissemination of disaster warning information and (5) community actions	Article 46, (2)
Disaster mitigation	(1) Implementation of spatial planning, (2) regulation of development, infrastructure development and building layout and (3) education, counselling and training	Article 47, (2)
**C. Post-disaster management**		
Emergency response	(1) Rapid assessment of location, damages and resources, (2) decision on the disaster emergency status, (3) search, rescue and evacuation of disaster victims or affected community, (4) basic needs fulfilment, (5) protection for the vulnerable group and (6) immediate recovery of essential facilities and infrastructure	Article 48
Rehabilitation	(1) Environmental improvement of post-disaster area, (2) rehabilitation of public facilities and infrastructure, (3) community housing repair assistance programme, (4) socio-psychological recovery, (5) health care services, (6) reconciliation and conflict resolution, (7) socio-economic and cultural recovery, (8) disaster recovery security and order, (9) recovery of government administration function and (10) public services recovery support function	Article 58, verse (1)
Reconstruction	(1) Facilities and infrastructure reconstruction, (2) reconstruction of community social facilities, (3) rebuilding sociocultural life of the community, (4) application of appropriate design and use of improved and disaster-resistant equipment, (5) participation of social institutions and organisations, private sector and community, (6) improvement of sociocultural and economic conditions, (7) improvement of public service functions and (8) improvement of essential public services	Article 59, verse (1)

Moreover, the Indonesian government of DM system allows NGOs or non-state actors to take a significant part. In fact, these actors play an important role in improving the DM system in Indonesia, namely to shift the Indonesia DM paradigm towards being more comprehensive and preventive (Simarmata & Suryandaru 2015).

The government of Indonesia has already established a scheme of DM planning (*Rencana Penanggulangan Bencana* [RPB]) which is managed by a ministry-level organisation called the National Agency for Disaster Management (or *Badan Nasional Penanggulangan Bencana* [BNPB]). Simarmata and Suryandaru (2015) believe that the DM planning is established by the Indonesia government to embrace a new paradigm of DM from a reactive to preventive and comprehensive approach to disasters. In this context, BNPB is responsible for DM action that includes planning development, analysing disaster risk, implementing spatial plans, educating and training people and implementing disaster risk management technical standards (Simarmata & Suryandaru, 2015).

In the local government, there are at least two government institutions that are responsible for promoting and implementing DRR, namely BPBDs and *Badan Perencanaan Pembangunan Daerah* (BAPPEDA – Development Planning Agency at Sub-National Level) (Mardiah, Lovett & Evanty 2015).

## Disaster budgeting in Indonesia

Budgeting is a process of planning and directing future programmes and expenditures in a department or organisation. The aim is to ensure that the programme can be implemented and that it is financially supported. The traditional approach of budgeting employs an incremental method in which budgeted expenditures and programmes heavily link with the budget of the previous year. In addition, the public fund is mainly allocated based on the size of departments or ministries rather than the need of each department or ministry. This traditional budgeting approach may not be suitable for disaster budgeting where allocation for disaster should be higher in the region with high disaster risk.

Indonesian DM Law No. 24/2007, Chapter VIII, regulates the funding mechanism and the management of aid. The law calls for a joint responsibility between central and local government to allocate DM funds. The law is only emphasised on emergency response funds; however, the pre- and post-disaster funds are not explicitly mentioned under the law. Further provisions have been regulated in Government Regulation (GR) No. 22/2008 concerning funding and disaster relief management.

The GR 22/2008 classified three types of DM funds, namely contingency funds, ready-to-use (or on-call) funds and social assistance funds (Das & Luthfi 2017). The contingency funds are budgeted for preventive programmes and major disasters that can happen in the future. The on-call funds can be used during the emergency phase and the social assistance funds are allocated for the early stage of post-disasters. These funds are budgeted in the national budget and sub-national budgets. Depending on the scale of disasters (national disaster or not), these funds are used collectively to support the DM programme in Indonesia.

The new DM system in Indonesia requires the allocation of the national budget for DM to go to the BNPB. In the context of regencies or districts (local government), each BPBD is the main actor of the local government DM budget. However, it is argued that Indonesian local governments do not have sufficient capacity to reduce disaster risks and respond to disasters (see, e.g. Djalante et al. 2015). In terms of financial resources, the BPBDs have a limited budget with which to finance their programme (see, e.g. Oktari et al. 2017). It can be associated with the role of the BAPPEDA as the key actor of budgeting and planning in the Indonesian local governments.

In the case of disaster relief, the budgeting of disaster programmes is challenging, full of uncertainty and potential dilemmas (Goodisman 1983). How much money should be spent and when it should be allocated in the relief programme or disaster agency cannot be precisely determined as it depends on disaster occurrences that are hard to predict (Goodisman 1983; Phaup & Kirschner 2010). The traditional role of accounting, where the concept of budgeting was created, does not recognise uncertain future events such as natural disasters (Sciulli 2017). Notwithstanding, this disaster budgeting involves many parties who may have different interests or who focus on optimising their own organisational and individual advantages (Hu et al. 2016).

Disaster expenditures can be classified into three groups, namely pre-disaster expenditures, emergency response expenditures and post-disaster expenditures. Pre-disaster expenditures are related to mitigation and preparedness programmes, for example, a disaster information brochure and disaster preparedness courses in schools. Emergency response expenditures can be defined as any cost incurred for helping victims when the disaster occurs. Post-disaster expenditures are expenditures associated with the recovery process. In the Indonesian case, more than half of Indonesian local governments allocate a lower mitigation budget compared to the international standard (Dartanto et al. 2017). Meanwhile, Oktari et al. (2017) found a limited budget portion of the DM agency is on the pre-disaster stage.

Governments need to be fully concerned disaster expenditures as there is a significant increase in disaster losses around the world. The rise of financial loss can be associated with population, environmental degradation and urbanisation (Ghesquiere & Mahul 2010). Moreover, emerging economies or developing countries are not able to provide sufficient funding for mitigation and prevention programmes as well as the proper procedures to utilise resources to anticipate an emergency situation (Ghesquiere & Mahul 2010).

Kusumasari et al. (2010) propose competence requirements of local governments in handling disasters. These authors conclude that local governments of developing countries and their communities do not have sufficient skills and expertise to respond to disasters. In terms of allocating budget for disasters, local governments face difficulty in shifting the budget to respond to disaster events because of complicated and rigid budgeting procedures (Labadie 2010).

Local governments might have allocated budget for disasters within several departments (social department, education department and health department) or only in a special agency that is responsible for disaster recovery. Research on this topic is limited and particularly with respect to how the government decides how much money needs to be allocated for disaster recovery and mitigation efforts. In the context of Indonesian local governments, Dartanto et al. (2017) found that one of the DB determinants is disaster risk, although half of the studied local governments have allocated a budget for disasters that is less than the international standard. Furthermore, Oktari et al. (2017) reveal that BPBD or the local DM agency budget is mainly routine expenses, that is, maintenance expenses, and the portion compared to the total budget of the Banda Aceh local government is very insignificant. The study also found that a DB is apparently formulated by using a similar approach (incremental budgeting) with the budget formulation of other working units or departments in the local government.

## Method

This study employed a quantitative approach to analyse the association between DB, disaster risk and the population of 23 districts in the Aceh province, northern Indonesia, for the periods of 2014, 2015 and 2016 (i.e. total observations = 66). The data were gathered from the local government budgets (or *Anggaran Pendapatan dan Belanja Daerah* [APBD], *Indeks Risiko Bencana* [or disaster risk index {DRI}]) and statistics reports published by the Indonesian Statistics Bureau. The operationalization of the variables can be found in Table 2.

**TABLE 2:** Variable definition and measurement.

Variables	Definition
DISBUGT	Disaster budget. Total of BPBDs annual budget
DRI	Disaster risk index or DRI (or *Indeks Risiko Bencana*). This index is computed by the National Agency for Disaster Management (or BNPB) http://inarisk.bnpb.go.id/irbi
Total budget	The total budget of the local governments
Population	Population of citizens in each local government area

DISBUGT, disaster budget; DRI, disaster risk index; BPBD, *Badan Penanggulangan Bencana Daerah*; BNPB, *Badan Nasional Penanggulangan Bencana.*

*Badan Penanggulangan Bencana Daerah* is a working unit of local government that is responsible for mitigating disaster impact, responding to the emergency situation and facilitating the process of recovery (Dartanto et al. 2017). The emergence of this body in each local government (provinces and districts) is the result of Law no. 24 (2007) which requires that each Indonesian local government has to establish a special body that focuses on disaster mitigation, the so-called BPBD. Each BPBD is expected to collaborate with each other BPBD to ensure effective responses towards disasters.

Furthermore, the BNPB calculates DRI every year and publishes it online in their website or reports. The DRI calculation can be used as an indicator to evaluate government efforts in reducing DRI especially in important economic growth areas in Indonesia (National Disaster Risk Management Agency 2016). The DRI is calculated based on three components, that is, hazard index, vulnerability index and capacity index for all potential disasters in each province and district in Indonesia. This index takes into consideration almost all potential disasters in Indonesia, namely earthquake, tsunami, volcano eruption, drought, landslide, bushfire, extreme weather and extreme sea wave and abrasion. The data are collected from each province and district in Indonesia.

In addition to secondary data, a serial survey and indirect interviews have been conducted. The informants were the budget preparers of the BPBD. A list of questions was sent via email to the budgeting or financial staff of three BPBDs, namely the Aceh province BPBD, Sabang and Aceh Besar BPBDs. This survey was conducted between June and September 2017. The result of the survey revealed an insight into disaster budgeting and a confirmation of the relationship between total budget and DB. The objective was to obtain detailed information about how the local government prepares the budget for disasters. We asked them to explain the budgeting process in their department, the key actors and the factors that determined the amount of the budget.

To examine the correlation between the variables, the Pearson’s chi-square test and the Pearson’s correlation test were performed. Both statistical formulas are able to evaluate the strength and the directions of the correlation. The statistical software used in this data analysis is the Statistical Package for the Social Sciences (SPSS). The expected results are a range of relationship levels between 0 and 1. The higher the correlation value, the stronger the relationship among the variables. Table 3 describes the classification of correlation levels and the interpretation.

**TABLE 3:** Rule of thumb for interpreting the size of a correlation coefficient.

Size of correlation	Interpretation
0.90–1.00 (−0.90 to −1.00)	Very high positive (negative) correlation
0.70–0.90 (−0.70 to −0.90)	High positive (negative) correlation
0.50–0.70 (−0.50 to −0.70)	Moderate positive (negative) correlation
0.30–0.50 (−0.30 to −0.50)	Low positive (negative) correlation
0.00–0.30 (0.00 to −0.30)	Negligible correlation

*Source*: Campbell, M.J. & Swinscow, T.D.V., 2009, *Statistics at square one*, 11th edn., BMJ Books, Southampton.

Later on, we conducted indirect interviews with senior officers by sending them a list of questions. Only three senior officers from the BPBDs returned their answers, that is, BPBA (Aceh province), BPBD Sabang and BPBD Aceh Besar. Based on their responses, the disaster budgeting process of the three local governments can be analysed.

## Overview of Aceh province, Indonesia

Indonesia is naturally a disaster-prone country. It has 34 provinces and Aceh is one of the provinces that was significantly affected by the Indian Ocean earthquake and tsunami disaster back in 2004. This made the region one of the most disaster-prone areas in Indonesia. According to the Indonesian Disaster Data and Information database (*Data dan Informasi Bencana Indonesia* [DIBI]), from 1815 to August 2018, a total of 1148 disasters have occurred in Aceh. The most frequent disasters are floods (479 occurrences) and fires (243 occurrences). Tsunamis have occurred four times, but the fatalities are higher than the other disasters, that is, 166 201 deaths. Thus, this province is among the provinces with one of the highest disaster risk levels in Indonesia. Additionally, the Aceh government has received special autonomy since 2008 from the central government. This has made Aceh as one of the provinces with the biggest budget in Indonesia.

### Ethical considerations

This article followed all ethical standards for research without direct contact with human or animal subjects.

## Results

Indonesia has adopted a decentralised government since 2001 where local governments enjoy an increasing authority to manage their resources. In terms of the budget, each local government preparing a budget will collaborate with the local parliament. The budgeting process is started with the proposed programme from each department and is compiled by the BAPPEDA of each local government. The BAPPEDA then selects which programme should be included in the local budget and which programme should be excluded. In other words, BAPPEDA is one of the key actors in local government budgeting which includes deciding how much money will be spent on disasters or BPBD.

The disaster-related programme can not only be found in BPBD, a relatively new department in the Indonesian local government structure (the BPBDs are transformed fire-fighter departments that have a recent extended role in disaster mitigation and prevention [Oktari et al. 2017]), but some disaster-related programmes are also allocated to the social department. These programmes are mostly for quick responses related to natural disasters, that is, a public kitchen and clothes for the victims. Meanwhile, most of the disaster-related programmes are budgeted in the BPBD.

This study demonstrates that local governments in the Aceh province allocated a relatively small amount of money for disasters, although the government budget is significantly bigger than most provinces in Indonesia. Total DB ranges from Indonesian Rupiah (IDR) 2 537 036 291.00 (or $195 157.00) to IDR 59 567 791 659.00 (or $4 582 138.00). The average value of DB is IDR 10 575 949 895.00 (or $813 534.00). The proportion of DB from the district budget is relatively very small. The highest DB portion is only 0.18, whereas the smallest portion is 0.85 with the average value being less than 1% (or 0.85). This portion is very small and this fact might indicate a weak motivation of the local governments in their programme of budgeting for a disaster.

Meanwhile, the DRI of the studied districts is relatively high (Table 4). The lowest DRI is 95 (medium risk) and the highest DRI is 211 (high risk). Additionally, the average DRI is 161 which indicates a high disaster occurrence risk in the studied areas. Thus, the studied local governments are located in regions that are highly exposed to natural disasters such as earthquake, flood, fire, drought, tsunami, volcano and landslide.

**FIGURE 1:**
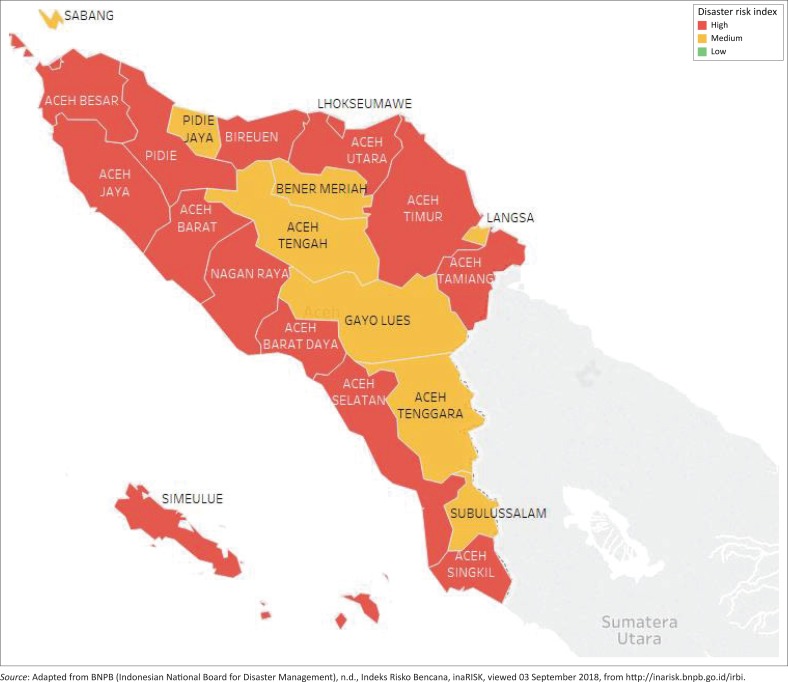
Disaster risk index of Aceh province.

**TABLE 4:** Descriptive analysis.

Items	Disaster risk index	Disaster budget (DB)†	Proportion of DB	Total District budget†	Population
Maximal value	211	59 567 791 659	4.18	2 714 861 998 954.00	593 492
Minimal value	95	2 537 036 291	0.18	522 534 046 526.00	33 215
Average	161	10 575 949 895	0.85	1 245 973 279 748.00	224 425

† , In Indonesian rupiah (IDR).

Furthermore, by employing Pearson’s chi-square test and the Pearson correlation test (Table 5), the DRI indicates a negligible correlation to DB (DISBUGT) (*r* = 0.005, *p* = 0.318). Similarly, the correlation between the DB and population showed an insignificant correlation (*r* = 0.030, *p* = 0.251). The same result is also found for the relationship between the DB proportion and DRI (*r* = −0.100, *p* = 0.402) and between the DB proportion and population (*r* = −0.170, *p* = 0.326). The only correlation that was confirmed is between DISBUGT and the total budget (*r* = 0.334, *p* = 0.023). Thus, the present study indicates that DB has a positive significant correlation with the total budget of the studied local governments. This can be interpreted as a larger DB being found in the local government with a bigger budget. In this case, local governments with a higher budget have a higher budget for disaster.

**TABLE 5:** Result of Pearson Chi-Square and Pearson Correlation test.

Variables	*p*	Pearson’s correlation value (*r*)
DISBUGT * DRI	0.318	0.005
DISBUGT * Population	0.251	0.030
DISBUGT * Total budget	0.023	0.334
DB proportion * DRI	0.402	−0.100
DB proportion * population	0.326	−0.170

DISBUGT, disaster budget; DRI, disaster risk index; DB, disaster budget.

To obtain a more detailed understanding of disaster budgeting, a survey that involved key officers in the budgeting of three BPBDs was conducted. The process of disaster budgeting was obtained based on the survey result. Firstly, the disaster budgeting (BPBD budget) process is similar to the budgeting in other departments. Each BPBD proposes an annual budget to the BAPPEDA. The BAPPEDA compiles the proposed budget from all departments and, later on, they make the local government budget. Because of financial constraints, the proposed budget from the BPBDs and other departments is evaluated and rationalised. Consequently, BPBDs mostly receive less budget than what they have proposed:

It follows a standard budgeting process regulated by the central government. Starting from determining long-term and short-term planning, followed by the proposed program. (Male, Senior staff, Budgeting Department at BPBD Aceh)

Secondly, the amount of local budget allocated for BPBDs is relatively small and not sufficient to cover the proposed BPBD programme. The reason could be that the district has a small total budget and the department is relatively new:

Our budget is still very limited. What we can do is to facilitate coordination when a disaster happens. The budget amount is not really optimal. (Male, Senior staff, Budgeting Department at BPBD Aceh Besar)

However, the province’s BPBD received a more ideal budget. The main reason is that the provincial BPBD has a more complex responsibility than the district BPBDs. It is responsible for any disaster that is categorised as a regional disaster. For example, an earthquake affects more than two or three districts, and therefore, it is categorised as a regional disaster; the Aceh province BPBD has to cover emergency and (partly) recovery programmes that are needed. Meanwhile, the district BPBDs are only responsible for coordination in disaster recovery and mitigation in their areas:

(The budget is) very ideal. In fact, the budget of BPBD of Aceh province is among the biggest compared to BPBD of other provinces in Indonesia. (Male, Senior staff, Budgeting Department at BPBD Aceh Besar)

Thirdly, the amount of the budget is mainly determined by the total budget of the local government. The portion seems too small compared to the budgets for other departments. This might indicate a lack of awareness and commitment of the local government as well as the political support of local parliament members on disaster mitigation and response issues.

The results of this study are consistent with those of Oktari et al. (2017) who found a limited portion of DB in the Banda Aceh local government, Indonesia. The previous study found that the DB is allocated mainly for routine administrative tasks of the BPBD. Notwithstanding, this disaster mitigation and preparedness programmes are very limited and the BPBD relied heavily on the support of their partners, that is, international NGOs and BNPB (Oktari et al. 2017; Putra & Matsuyuki 2019). Another study, for example, Intarti et al. (2013), reveals that the government of Jakarta, the capital of Indonesia, allocates a minimum budget of 1% of the total government budget for the BPBD although the government has integrated its DM fund with the government in a short-term planning document.

The result of the present research does not confirm the results of the study by Dartanto et al. (2017) where it was found that the Indonesian local governments studied are taking into account the disaster risk when allocating their DBs. The reason for this divergent result could be that local governments in Aceh have different budget priorities and capacities. Additionally, the financial regulation of minimum allocation for the education and health sectors makes the budget and its allocation more complicated. As a result, the allocation for BPBD in many cases will be determined at the end of the budgeting process.

Furthermore, the budget procedures and regulations for BPBDs are similar to other departments or local government institutions. The main determinant of the DB could be the total budget of the local government instead of the potential disaster risk of each region or district. In fact, the BPBD programme seems to not be at the top of the list of priorities in local government budgeting. The main possible explanation is that BPBD is a relatively young and new government institution that leads to less concern, trust and respect from the local government structure (Simarmata & Suryandaru 2015).

The other reason could be a lack of commitment and capacity of the local governments. This has also been demonstrated in the study by Mardiah et al. (2015) which evaluates the regulatory and institutional frameworks of DRR in Indonesia. They report a lack of commitment and capacity of local authorities to adopt the DRR agenda into their development programme. The disaster-related programme could be considered as expenditure without real value rather than an investment to avoid significant losses because of disasters (Anantasari et al. 2015).

The local government should increase the budget proportion for disaster-related programmes or BPBD. One way to do that is to make it obligatory to have a minimum allocation for a disaster programme. For example, the local governments could be required to allocate at least 5% of their total budget for disaster mitigation-related programmes. This requires a revision of the GR 22/2008, which states that an on-call budget for DM can be provided by local governments, instead of having to be provided by central government (Mardiah et al. 2015). The on-call budget should be allocated by local governments along with the BPBD budget on a regular basis (Mardiah et al. 2015). The local governments should be facilitated and encouraged to provide sufficient allocation for DRR programmes (Das & Luthfi 2017).

More importantly, the capacity of local government and the knowledge of the senior officers of BPBD in DM should be improved so that they can prepare an effective disaster programme and budget (Kusumasari et al. 2010). As aforementioned, in the Aceh province, the BPBDs are actually former fire departments. The senior officers do not have adequate skills or educational background in DM (Oktari et al. 2017). Thus, formulating and proposing an appropriate DM fund, programmes and an integrated DB are problematic and difficult. Ideally, the budget should take into account not only the post-disaster programmes (reconstructions, recovery programme), but also publication and preventive actions. The policy and DRR concept should be adopted and budgeted by other departments in local governments. In addition, the involvement of community leaders and community participation in DM planning is imperative (Gianisa & Le De 2018). Such an integrative mechanism is necessary to ensure that all preventive efforts can be undertaken. For example, a pre-disaster programme such as hospital disaster construction and student knowledge on disaster should be budgeted for in the health department and education departments.

Lastly, a DB should be classified based on its function rather than the scope of disasters. Disaster preventive programmes, which are not urgent and can be arranged over a relatively long period of time, can be allocated by each BPBD. Meanwhile, the BNPBs focus more on the impact of disasters, recovery and reconstruction as well as internalising the disaster risk management concept into government daily operations. Thus, it is suggested that each local government improves the knowledge and skills of BPBDs staff with the purpose of equipping them in creating an innovative programme to anticipate and respond to the natural disaster effectively (Putra & Matsuyuki 2019).

## Conclusion

Although there has been a greater call on improving the role of local governments in DM, including DM financing, the present study finds a contradictory situation. The allocated DB in BPBDs in the Aceh province, northern Indonesia, is relatively small compared to the local government budget (on average less than 1%). The present study demonstrates statistically an insignificant correlation between disaster budgeting and disaster risk index (DRI) in the local governments of the Aceh province, northern Indonesia. However, there is a significant positive correlation between DB and total budget of the local government. Hence, it can be concluded that the DB is not formulated based on the possibility of a disaster occurrence in the districts or on the population, but rather it is based on the total amount of budget of each local government (proportionately). In addition, this study reveals that the DB is prepared in a similar manner to other department budgets. The main actors are BAPPEDA and the local government budgeting team as they decide the approved budget for all departments. The possible determinants of DBs could be the total amount allocated to local government budgets, politics and the commitment of government and legislation on DRR, the complexity and scope of BPBD duties and accountability of previous budget spending. These potential determinants should be tested in further research. This study has some limitations, namely, a small number of data samples for analysis, a short period of observation and minimal data analysis. It is imperative to re-assess the correlation between DB and DRI in bigger samples or to examine the determinants of the DB in the Indonesian or cross-country setting. Therefore, it is imperative to conduct similar research using a bigger sample, for example, all local governments throughout Indonesia.
